# The Snail transcription factor CES-1 regulates glutamatergic behavior in *C*. *elegans*

**DOI:** 10.1371/journal.pone.0245587

**Published:** 2021-02-02

**Authors:** Lidia Park, Eric S. Luth, Kelsey Jones, Julia Hofer, Irene Nguyen, Katherine E. Watters, Peter Juo

**Affiliations:** 1 Department of Developmental, Molecular and Chemical Biology, Tufts University School of Medicine, Boston, Massachusetts, United States of America; 2 Graduate Program in Cell, Developmental and Molecular Biology, Graduate School of Biomedical Sciences, Tufts University School of Medicine, Boston, Massachusetts, United States of America; 3 Department of Biology, Simmons University, Boston, Massachusetts, United States of America; 4 Graduate Program in Neuroscience, Graduate School of Biomedical Sciences, Tufts University School of Medicine, Boston, Massachusetts, United States of America; Northwestern University, UNITED STATES

## Abstract

Regulation of AMPA-type glutamate receptor (AMPAR) expression and function alters synaptic strength and is a major mechanism underlying synaptic plasticity. Although transcription is required for some forms of synaptic plasticity, the transcription factors that regulate AMPA receptor expression and signaling are incompletely understood. Here, we identify the Snail family transcription factor *ces-1* in an RNAi screen for conserved transcription factors that regulate glutamatergic behavior in *C*. *elegans*. *ces-1* was originally discovered as a selective cell death regulator of neuro-secretory motor neuron (NSM) and I2 interneuron sister cells in *C*. *elegans*, and has almost exclusively been studied in the NSM cell lineage. We found that *ces-1* loss-of-function mutants have defects in two glutamatergic behaviors dependent on the *C*. *elegans* AMPA receptor GLR-1, the mechanosensory nose-touch response and spontaneous locomotion reversals. In contrast, *ces-1* gain-of-function mutants exhibit increased spontaneous reversals, and these are dependent on *glr-1* consistent with these genes acting in the same pathway. *ces-1* mutants have wild type cholinergic neuromuscular junction function, suggesting that they do not have a general defect in synaptic transmission or muscle function. The effect of *ces-1* mutation on glutamatergic behaviors is not due to ectopic cell death of ASH sensory neurons or GLR-1-expressing neurons that mediate one or both of these behaviors, nor due to an indirect effect on NSM sister cell deaths. Rescue experiments suggest that *ces-1* may act, in part, in GLR-1-expressing neurons to regulate glutamatergic behaviors. Interestingly, *ces-1* mutants suppress the increased reversal frequencies stimulated by a constitutively-active form of GLR-1. However, expression of *glr-1* mRNA or GFP-tagged GLR-1 was not decreased in *ces-1* mutants suggesting that *ces-1* likely promotes GLR-1 function. This study identifies a novel role for *ces-1* in regulating glutamatergic behavior that appears to be independent of its canonical role in regulating cell death in the NSM cell lineage.

## Introduction

Changes in the strength of glutamate signaling during synaptic plasticity underlies information processing and storage in the brain [1]. Dysregulation of glutamatergic synapse development and function contributes to several neurological diseases including autism spectrum disorders, epilepsy, depression and Alzheimer’s Disease [2–6]. Regulation of AMPA-type glutamate receptor levels or signaling at synapses is a major mechanism that contributes to changes in synaptic plasticity such as long-term potentiation, long-term depression, and homeostatic plasticity [7]. Although transcription is required for some forms of plasticity, such as synaptic homeostasis [8, 9], only a few transcription factors are known to regulate AMPA receptor expression or signaling, including Nuclear respiratory factor-2 (Nrf-2) [10], Specificity protein 4 (Sp4) [11], Pro-myelocytic leukemia protein (PML) [12] and Serum response factor (SRF) [13].

We took advantage of a simple glutamatergic mechanosensory behavior in *C*. *elegans* to identify conserved transcription factors that regulate glutamatergic signaling. Light touch to the nose of the worm activates several neurons including the glutamatergic sensory neuron pair ASH [14–17]. ASH in turn activates downstream command interneurons that express the AMPA receptor GLR-1 that ultimately results in a locomotion reversal [18–21]. Using an optogenetic version of this behavior, we performed an RNAi screen of conserved transcription factors and identified several candidates, including the cell death specification gene *ces-1*. CES-1 belongs to the Snail family of zinc finger transcription factors that regulates cell polarity, cell proliferation, and cell death during development in *C*. *elegans*, *Drosophila*, and mammals [22–24]. For example, Snail family transcription factors repress genes that promote cell polarity and cell adhesion, resulting in epithelial-to-mesenchymal transitions (EMTs). During normal development, EMTs enable migratory cell behavior critical for tissue and organ development; however, dysregulation of Snail family transcription factors and EMTs can lead to tumorigenesis and metastasis [23, 25]. In *Drosophila*, Snail family transcription factors *snail*, *escargot*, and *worniu* regulate neuroblast polarity by controlling asymmetric cell division [26, 27]. In mammals, Snail1 inhibits cell proliferation by repressing cyclin D2 [28], and the Snail family member Slug inhibits apoptosis by repressing transcription of the BH3 only protein PUMA [29]. More recently, there has been growing interest in the Scratch subfamily of Snail family transcription factors. Unlike the Slug subfamily, Scratch subfamily transcription factors appear to have neuronal-specific expression and function, promoting neuronal EMTs, differentiation, and cell survival during development [30–34]. CES-1 was originally thought to be most similar to the Scratch subfamily transcription factors based on sequence and function [35], however a subsequent study suggests that unlike mammalian SCRATCH, CES-1 may also function outside the nervous system [36].

In *C*. *elegans*, CES-1 regulates asymmetric cell division [37–39], cell proliferation [36], and cell death [35, 40] in specific cell lineages. A role for Snail family genes in cell death was originally discovered in *C*. *elegans*, where *ces-1* was shown to selectively regulate cell death in the NSM neuro-secretory motor neuron and the I2 interneuron cell lineages [40]. Specifically, two NSM neuroblasts divide asymmetrically to generate two daughter cells. One daughter cell differentiates into a serotonergic motor neuron whereas the other sister cell undergoes programmed cell death. *ces-1(n703)* gain-of-function (gf) mutations prevent the death of NSM and I2 sister cells [35, 40]. NSM sister cell death is dependent on increased transcription of the BH3-only EGL-1 protein, which promotes cell death upstream of the canonical cell death pathway [35, 41]. CES-1 binds to Snail binding E-box sites in a *cis*-regulatory element downstream of the *egl-1* coding region and antagonizes the function of basic helix-loop-helix (HLH) transcription factors HLH-2 and HLH-3, which activate *egl-1* transcription via the same binding sites [41]. With the exception of one study, which showed that *ces-1* mutants enhance cell division defects of cell cycle mutants in several lineages [36], studies of *ces-1* have been focused on its role in the NSM and I2 cell lineages.

Here we report a novel role for *ces-1* in regulating glutamatergic behavior in *C*. *elegans*. This function does not involve the death of neurons directly involved in the glutamatergic behaviors examined and appears to be unrelated to the known role of *ces-1* in regulating cell death in the NSM lineage.

## Materials and methods

### Strains

All strains were maintained at 20 degrees Celsius [42]. N2 Bristol was used as wild type.

AQ2235: *ljIs114 (Pgpa-13*::*FLPase*, *Psra-6*::*FTF*::*ChR2*::*YFP) lite-1(ce314) X* (gift from William Schafer)

TU3401 *uIs69 (Pmyo-2*::*mCherry*, *Punc-119*::*sid-1) sid-1(pk3321*) *V*

FJ1300: *nuIs25 (Pglr-1*::*glr-1*::*GFP*, *lin-15(+)) lin-35(n745) I; uIs69 (Pmyo-2*::*mCherry*, *Punc-119*::*sid-1) sid-1(pk3321*) *V; ljIs114 (Pgpa-13*::*FLPase*, *Psra-6*::*FTF*::*ChR2*::*YFP) lite-1(ce314) X*

KP4: *glr-1(n2461) III*

FJ465: *ric-4(md1088) V*

FX01036: *ces-1(tm1036) I*

MT8704: *ces-1(n703n1434) I*

MT2557: *ces-1(n703) I*

FJ1577: *ces-1(n703) I; glr-1(n2461) III*

ESL4: *ces-1(tm1036) I; pzEx448 (Pglr-1*::*ces-1; Pmyo-2*::*mCherry)*

KP2006: *nuIs80 (Pglr-1*::*GLR-1(A/T))* (gift from Joshua Kaplan)

FJ1511: *nuIs80; ces-1(tm1036) I*

FJ1500: *nuIs80; ces-1(n703n1434) I*

FJ1640: *nuIs80; ces-1(tm1036) I; pzEx448 (Pglr-1*::*ces-1; Pmyo-2*::*mCherry)*

FJ1755 *ces-2(bs213) I* (gift from Barbara Conradt)

VC1689: *ces-2(gk1020) I*

FJ1047: *pzIs29 (Pglr-1*::*NLS*::*GFP*::*LacZ) X* [43]

FJ1488: *pzIs29; ces-1(tm1036) I*

FJ1489: *pzIs29; ces-1(n703n1434) I*

FJ1490: *pzIs29; ces-1(n703) I*

FJ1398: *pzIs29; unc-42(e270) V*

VM484: *akIs3* (*Pnmr-1*::*GFP; lin-15(+)) V* [44]

FJ1658: *akIs3 (Pnmr-1*::*GFP; lin-15(+)) V; ces-1(tm1036) I*

FJ464: *pzIs12 (Pglr-1*::*HA*::*GLR-1*::*GFP) II* [45]

FJ1501: *pzIs12 II; ces-1(tm1036) I*

FJ1554: *pzIs12 II; ces-1(n703gf) I*

*bcSi66 (Pces-1*::*ces-1*::*mNeonGreen) II* (gift from Barbara Conradt) [39]

### Plasmids and transgenes

#### *Plasmid Pglr-1*::*ces-1* (FJ#139)

*ces-1* genomic DNA sequence was PCR amplified from pBC510 (*Pces-1*::*ces-1*::*yfp*) (gift from Barbara Conradt) [37] as a template. Nhe I and Sac I restriction sites were added using oligos flanking the *ces-1* coding region and subcloned into pV6 (*Pglr-1* promoter in pPD49.26) using Nhe I and Sac I. The final construct was verified by sequencing. Transgenic strains were created using standard microinjection techniques. FJ#139 was injected at 0.1 ng/μL together with the coinjection marker *Pmyo-2*::*mCherry* to create *pzEx448*. *bcSi66* (*Pces-1*::*ces-1*::*mNeonGreen*) is a single copy transgene inserted into Chromosome II using MosSCI, that has been previously described [39]. Briefly, Wei et al. (2020) created this translation *ces-1* reporter using the genomic AflII-SpeI fragment (~9.3 kb) from cosmid F43G9 (via pBC510 and pBC1448) and inserted mNeonGreen in-frame at the C-terminus. This transgene contains the genomic *ces-1* coding region (~2.1 kb) with ~2.5 kb of 5’UTR upstream of the start codon and ~4.7 kb genomic sequence downstream of the stop codon [39].

### Optogenetic RNAi screening

RNA interference (RNAi) was performed using the FJ1300 strain which expresses channelrhodopsin-2 (ChR2) in ASH sensory neurons [*lite-1 (ce314)*, *ljIs114 (gpa-13p*::FLPase, *sra-6p*::*FTP*::ChR2::YFP)] [46] in a genetic background that enhances neuronal RNAi [*lin-35(n745); uIs69 (Punc-119*::*sid-1); sid-1(pk3321)*] [47]. An RNAi sub-library [48, 49] targeting 318/330 transcription factors was curated from a list of putative transcription factor genes with human orthologs (OrthoList) [50]. Bacteria were maintained in the presence of 50 μg/ml ampicillin (Sigma #A9518) for plasmid selection. One generation RNAi was performed as follows: 1 mL overnight cultures of bacteria carrying RNAi plasmids were grown at 37 °C in Luria broth containing 50 μg/ml ampicillin. 30 μl of all-*trans* retinal (ATR, Sigma #R2500) was dissolved in ethanol to a final concentration of 100 μM and added to the cultures. Individual cultures were spotted in quadruplicate on 24-well nematode growth medium (NGM) agar plates containing 50 μg/ml carbenicillin (Sigma #C1389) and 5 mM isopropyl-β-D-thiogalactopyraniside (Sigma #PHG0010). Plates were allowed to dry for 1 hour under a hood and then either used immediately or wrapped in aluminum foil to protect the light-sensitive ATR and stored at 4 degrees Celsius for later use. Two gravid adult FJ1300 worms were placed in each well of the spotted NGM plates for ~24 h. The adults were removed and the eggs were allowed to develop to the L4 larval stage (~2–3 days). For optogenetic screening, each clone was screened in quadruplicate with the experimenter blinded to the identity of the RNAi clone. Individual wells were illuminated with 1 s pulses of blue light (0.47 mW/mm^2^) from a mercury bulb filtered through a GFP excitation filter under 32x total magnification on a Leica MZ16F microscope. Locomotor reversals (scored as positive if the distance reversed was greater than the distance from the nose to the terminal pharyngeal bulb) observed during or immediately after illumination were counted as responsive, and wells were scored on a 3-point scale based on an estimate of how many worms were responsive in each well. General locomotor activity was also noted and scored on a 3-point scale. Clones for which knockdown resulted in an impaired stimulated reversal response but wild type-like gross locomotor activity in 3 out of 4 wells for each experiment were deemed impaired for optogenetic reversal activity. These positive hits were rescreened in quadruplicate with the same criteria to identify candidates for further study.

### Aldicarb assay

The day before assaying, standard NGM plates with aldicarb (1mM, Sigma) were made and seeded with OP50 and dried by a Bunsen burner flame. 20 young adults of each genotype were plated onto aldicarb plates, with the experimenter blinded to the genotypes. At each time point, the ability of each worm to move was assayed by prodding the anterior of the worm using a standard platinum wire worm pick. Worms were assayed every 15–30 minutes over the course of 3 hours for a response (i.e., any body movement upon stimulation). Results were recorded as percent paralyzed over time. This was repeated over several days of trials. Sample number and statistical analysis (Prism 7, GraphPad) are described in the figure legends.

### Nose touch assay

For the nose touch response assay, standard NGM agar plates were seeded with OP50 (diluted 1:10) and dried near a Bunsen burner flame for 30 minutes-1 hour or until dried. The following day, individual young adult worms were transferred to this plate, with the experimenter blinded to genotypes. These worms were manually stimulated on the nose with an eyelash attached to a wooden dowel. The eyelash was placed at a 90-degree angle to the direction of worm movement and the reversal response recorded out of 10 total touches. This was repeated over several days of trials. Sample number and statistical analysis (Prism 7, GraphPad) are described in the figure legends.

### Spontaneous reversals assay

For spontaneous reversals, unseeded standard NGM agar plates were prepared the day before assaying. The following day, plates were dried in a fume hood for 5–20 minutes to remove excess moisture from plates. Because different levels of plate wetness introduces variability into the spontaneous reversal assay, assay plate quality was determined based on the spontaneous reversal frequencies observed for 2–3 wild type young adult animals. Plates were selected for the experiment when the average response of wild type worms was approximately ~5 reversals/minute, as previously described [45, 51]. The experimenter was blinded to the individual genotypes prior to assaying. For assaying, single young adults were picked with oil onto the assay plate and allowed to acclimate for 2 minutes. The number of spontaneous reversals was counted over the following 5 minutes and recorded as average reversals per minute. This was repeated several times across all genotypes and over several days of trials. Sample number and statistical analysis (Prism 7, GraphPad) are described in the figure legends. 7.

### Fluorescence microscopy

Imaging was performed with a Carl Zeiss Axiovert M1 microscope with 100x Plan Aprochromat objective. Images were taken with a Hamamatsu Orca-ER charge-coupled device and processed with MetaMorph, version 7.1 software (Molecular Devices). L4 hermaphrodites were used for all imaging. Animals were paralyzed with 30 mg/mL 2, 3-butanedione monoxamine (BDM, Sigma-Aldrich) for 5 minutes before imaging. For quantitative VNC imaging, maximum intensity Z-series stacks, 1 μm total thickness, were taken from the anterior VNC. FluoSphere fluorescent beads were used to normalize image fluorescence intensity. Line scans were made using MetaMorph (v6.0) and analyzed using IgorPro (v5) and custom-written software [52]. Constant exposure settings were used across genotypes and across all imaging days. Cell counts of GLR-1-expressing neurons marked with *Pglr-1*::NLS-GFP-LacZ (*pzIs29*), NMR-1-expressing neurons marked with *Pnmr-1*::GFP (*akIs3*), ASH neurons stained with DiI and ASH neurons marked with ChR2::YFP (*ljIs114*) were done manually by focusing through the worm head using a 63X objective.

### DiI imaging

Worms were washed from NGM plates with 1 ml M9 buffer into microfuge tubes, spun at 400 x *g* for 2 minutes, and resuspended in 1 mL M9. 5 μL of 2 mg/mL DiI (1,1′-Dioctadecyl-3,3,3’,3’-tetramethylindocarbocynanine perchlorate)(Sigma #468495) in dimethyl formate was added to each tube and incubated overnight with rocking. Worms were washed twice with M9, paralyzed with 30 mg/mL 2, 3-butanedione monoxamine (Sigma-Aldrich), and transferred onto 2% agarose pads. ASH was identified by position relative to other labeled amphid sensory neurons, and cell counts were done manually by focusing through the worm head using a 63X objective.

### Real-time quantitative PCR

Total RNA was isolated from 15 6-cm plates of mixed-stage animals per genotype, with 3 biological replicates per genotype. RNA was extracted with Trizol (Invitrogen) and a RNeasy Fibrous Tissue Mini kit (Qiagen). cDNA was prepared using Superscript III Reverse Transcriptase kit (Invitrogen). Real-time PCR was performed using SYBR Green Master Mix on a BioRad CFX96 Real-Time System C1000 Thermal Cycler machine. ΔΔCt was calculated for mRNA levels compared to 2 reference genes, *act-1* and *ama-1*, as previously described [43]. Results described are from 4 independent experiments. Sample number and statistical analysis (Prism 7, GraphPad) are described in the figure legends. The following primers were used: act-1F: CCAGGAATTGCTGATCGTATGCAGAA, act-1R: TGGAGAGGGAAGCGAGGATAGA, ama-1F: ACTCAGATGACACTCAACAC, ama-1R: GAATACAGTCAACGACGGAG, glr-1F: CCGTTTAAACTTGCATTTGACC, glr-1R: ACAGACTGCGTTCACCATGT.

## Results

### *ces-1* mutants have defects in a glutamatergic mechanosensory reflex behavior

We developed an optogenetic, behavioral RNAi screen to identify novel transcription factors (TFs) that regulate glutamatergic behavior. We used a simple, glutamatergic mechanosensory reflex behavior called the nose-touch response to screen for genes involved in glutamatergic signaling. In this avoidance response, mechanical stimulation (i.e., light touch to the nose with an eyelash) activates a pair of glutamatergic sensory neurons, ASH, in addition to other neurons [14–17]. Stimulation of ASH results in activation of downstream command interneurons (i.e., AVA, AVD, AVE) via GLR-1 AMPA receptors [18–21]. The command interneurons, in turn, signal to motor neurons that activate body wall muscle, resulting in backward locomotion away from the stimulus [53]. Mutants lacking the presynaptic vesicular glutamate transporter (VGLUT) *eat-4* [54] or glutamate receptor *glr-1* [18, 19] exhibit strong defects in the nose-touch response. Expression of Channelrhodopsin-2 (ChR2) specifically in ASH neurons [46, 55], allows us to stimulate ASH with blue light and observe reversal responses (optoASH assay) in a large population of worms. To enrich for genes that function in the nervous system, we expressed ChR2 in ASH in a genetic background that enhances the efficiency of neuronal RNAi [47]. We knocked down individual TF genes from an RNAi library of 318/330 putative *C*. *elegans* transcription factors predicted to have mammalian orthologs [50] and used the optoASH assay to screen for conserved TFs required for the glutamate-dependent reversal behavior. We identified the Snail family-related zinc finger transcription factor *ces-1* in this RNAi screen (Material and Methods). *ces-1* was originally identified in *C*. *elegans* as a regulator of cell death that functions upstream from the canonical cell death pathway consisting of *ced-9*/Bcl2, *ced-4*/APAF1 and *ced-3*/caspase [35]. However, *ces-1* is not broadly involved in cell death, but instead is only known to function as a specific cell death regulator of sister cells of the serotonergic NSM neuron and the I2 pharyngeal interneuron [35, 40]. While *ces-1(n703)* gain-of-function mutants prevent NSM and I2 sister cell deaths, *ces-1* loss-of-function mutants have no obvious cell death defects, perhaps due to redundancy [40].

**Fig 1:**
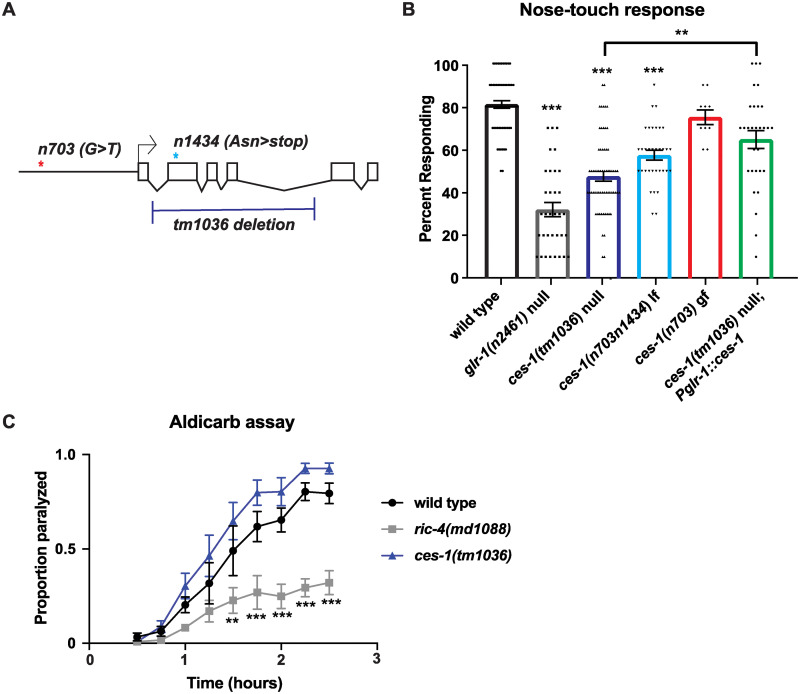
CES-1 regulates the nose-touch response, a GLR-1-dependent mechanosensory reflex behavior. (A) Diagram depicting *ces-1* and alleles used in this study. Boxes represent exons, V lines represent introns, straight line represents the upstream promoter region. The red asterisk marks the location of the G>T nucleotide change found in the *n703* gf allele and the blue asterisk marks the location of the Asn>stop codon change in the *n1434* allele. The bracket represents the region deleted in the tm1036 allele. (B) Nose touch response of wild type, *glr-1* and *ces-1* mutants, and *ces-1* mutants expressing *Pglr-1*::*ces-1* to mechanical stimulation to the tip of the nose with an eyelash. Results are shown as average response over 10 trials per individual worm. N = 66 animals for wild type, N = 34 for *glr-1*, N = 66 for *ces-1*(*tm1036)*, N = 34 for *ces-1(n703n1434)*, N = 10 for *ces-1(n703)*, and N = 30 for *ces-1(tm1036); Pglr-1*::*ces-1*. (C) Rate of paralysis after treatment with aldicarb for wild type, *ric-4/*SNAP-25, and *ces-1* mutants. Results are shown as the proportion paralyzed on plates with 200 ng/mL aldicarb tested at 15-minute intervals for 3 hours. N = 6 plates per genotype with 15–23 animals/plate. Error bars represent SEM. Asterisks above the bar indicate values that differ significantly from wild type. “ns” indicates no statistically significant difference from wild type (p>0.05). One-way ANOVA with Dunnett’s multiple comparisons test (B) or two-way ANOVA with multiple comparisons (C) was used to compare means. **p<0.005, ***p<0.0005.

We confirmed that *ces-1* is involved in the glutamatergic nose-touch response by measuring the response of two *ces-1* loss-of-function (lf) alleles, *n703n1434* and *tm1036*, and one *ces-1* gain-of-function (gf) allele, *n703* [35, 36, 40]. Importantly, these nose-touch assays were performed with an eyelash in the absence of ChR2 expression or the neuronal RNAi-enhancing mutant background used for the optogenetic RNAi screen. The *n703* gf allele consists of a G>T point mutation in the 5’ cis-regulatory region of *ces-1* about 600 bp upstream of the transcriptional start site and near where the negative *ces-1* regulator CES-2 binds [35]. The *n703n1434* lf allele suppresses the *n703* gf allele and contains a point mutation (Asn40Stop) that results in a premature stop codon [35], whereas the *tm1036* lf allele consists of a 1.2 kb deletion that eliminates the second and third DNA-binding Zn finger domains, likely resulting in a functional null mutant [36] (Fig 1A). We found no difference in the nose-touch response of *ces-1(n703)* gf mutants compared to wild-type animals, however, both *ces-1(tm1036)* and *ces-1(n703n1434)* lf mutants had reduced responses in the nose-touch assay (Fig 1B). Expression of wild-type *ces-1* cDNA in GLR-1-expressing neurons resulted in partial rescue of the nose-touch defect observed in *ces-1*(*tm1036*) null mutants, suggesting that CES-1 may function, in part, by acting in GLR-1-expressing neurons. These data support our *ces-1* RNAi knockdown results and indicate that CES-1 regulates glutamatergic mechanosensory nose-touch behavior.

### *ces-1* mutants have normal numbers of ASH sensory neurons

*ces-1* is known to regulate the cell death of NSM neuron sister cells by repressing transcription of the pro-apoptotic BH3 domain protein EGL-1 [37, 40]. In wild type animals, NSM neuron sister cells undergo cell death, whereas in *ces-1(n703)* gf mutants, the NSM sister cells are protected from cell death. Since *ces-1* is known to regulate cell death and laser ablation of ASH sensory neurons results in defective nose-touch responses [16], we tested if the nose-touch defects observed in *ces-1* lf mutants was due to ectopic cell death of ASH sensory neurons. We counted the number of ASH sensory neurons in the head using a strain that expresses a YFP reporter in the ASH neuron pair. We found no change in the number of ASH neurons in *ces-1(tm1036)* lf mutant animals compared to wild-type controls (Number of ASH cells (Mean ± SEM): WT: 1.96 ± 0.03 n = 29; *ces-1(tm1036)*: 2.0 ± 0, n = 42. p>0.05, unpaired Student’s *t* test). In addition to counting the number of ASH sensory neurons, we tested if ASH cell body location and process development were normal in *ces-1* mutants. ASH neurons extend a dendrite to the tip of the nose. The trajectory and morphology of these dendrites, which diffusely express YFP fluorescence throughout the ASH processes, appear grossly normal in wild type and *ces-1(tm1036)* mutants. Furthermore, the dendrites are exposed to the external environment at the tip of the nose and incubation of worms with the lipophilic fluorescent dye DiI results in backfilling of the dendrite and cell body of ASH and several other amphid sensory neurons exposed to the external environment [56]. Based on DiI staining, the number of ASH neurons in *ces-1*(*tm1036*) lf mutants was also unaltered compared to wild type (Number of ASH cells (Mean ± SEM): WT: 2.0 ± 0, n = 18; *ces-1(tm1036)*: 2.0 ± 0, n = 20, p>0.05, unpaired Student’s *t* test). These data suggest that in *ces-1* lf mutants the number, position, and morphology of ASH dendrites and cell bodies appear grossly normal, and that the nose-touch defect observed in *ces-1* lf mutants is not due to ectopic cell death of ASH neurons.

### *ces-1* mutants have wild-type neuromuscular junction function

A robust reversal response to the mechanical nose-touch stimulus requires normal neuromuscular junction and muscle function. Thus, we tested whether the defect in the nose-touch response observed in *ces-1* mutants was due to a defect in cholinergic transmission at the neuromuscular junction (NMJ). We measured NMJ function using the aldicarb-paralysis assay. After presynaptic release, acetylcholine (ACh) is broken down by acetylcholinesterase in the synaptic cleft. Aldicarb is a cholinesterase inhibitor and ACh accumulates in the synaptic cleft at the NMJ in worms exposed to the drug, leading to activation of ACh receptors and muscle paralysis over time. Mutations that affect presynaptic vesicle release or postsynaptic muscle function have defects in the aldicarb-paralysis assay [57–59]. We found that *ces-1(tm1036)* mutants paralyze with a similar time course as wild type animals when exposed to aldicarb (Fig 1C). In contrast, we found that mutants lacking *ric-4*/SNAP25, a SNARE broadly required for synaptic vesicle fusion, paralyze more slowly than wild-type animals. This result suggests that *ces-1* mutants have normal NMJ signaling. These data also suggest that *ces-1* mutants do not have a general defect in synaptic transmission, but rather, exhibit a relatively specific defect in glutamatergic signaling.

### *ces-1* mutants have defects in GLR-1-dependent spontaneous locomotion reversals

**Fig 2:**
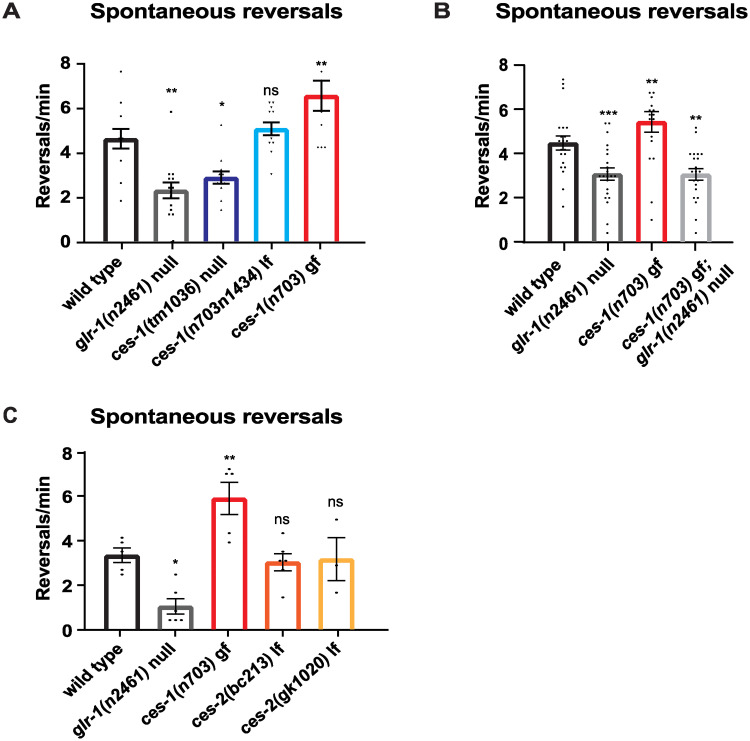
CES-1 regulates spontaneous reversals, a GLR-1-dependent locomotion behavior. (A-C) Average rate of spontaneous reversals measured on NGM standard plates in the absence of food over 5 minutes for the indicated genotypes. (A) Reversal frequency measured for wild type, *glr-1* null, *ces-1* lf and *ces-1* gf mutants. N = 12 animals for wild type, N = 14 for *glr-1*, and N = 12 for each of the *ces-1* alleles. (B) Reversal frequency measured for wild type, *glr-1* null, *ces-1* gf, and *ces-1* gf; *glr-1* null mutants. N = 21 animals for wild type, N = 22 for *glr-1*, N = 18 for *ces-1(n703)*, and N = 21 for *ces-1(n703); glr-1*. (C) Reversal frequency measured for wild type, *glr-1* null, *ces-1* gf, and *ces-2* lf mutants. N = 5 for wild type, N = 6 for *glr-1*, N = 5 for *ces-1(n703)*, N = 6 for *ces-2(bc213)*, and N = 3 for *ces-2(gk1020)*. Error bars represent SEM. Asterisks above the bar indicate values that differ significantly from wild type. “ns” indicates no statistically significant difference from wild type (p>0.05). One-way ANOVA with Dunnett’s multiple comparisons test was used to compare means. *p<0.05, **p<0.005, ***p<0.0005.

In order to test if *ces-1* specifically affects the nose-touch response or regulates other glutamatergic behaviors, we analyzed spontaneous locomotion reversals, in *ces-1* mutants. The frequency of spontaneous reversals during *C*. *elegans* locomotion is regulated by the level of glutamatergic signaling [60]. Mutants with decreased glutamatergic signaling, such as mutants lacking the vesicular glutamate transporter (VGLUT) *eat-4* [52, 60], the postsynaptic AMPA receptor *glr-1* [20, 44], or mutants with reduced levels of synaptic GLR-1 [45], exhibit decreased frequencies of spontaneous reversals. Conversely, animals with increased glutamatergic signaling, such as worms with increased synaptic surface levels of GLR-1 [61–63], or those expressing a constitutively-active form of GLR-1, GLR-1(A/T) [60], exhibit increased spontaneous reversal frequencies. We found that *ces-1(tm1036)* null mutants exhibit decreased reversal frequencies, whereas *ces-1(n703n1434)* lf mutants had wild type reversal frequencies (Fig 2A). The lack of a reversal frequency defect in *ces-1 (n703n1434)* lf compared to *ces-1 (tm1036)* null mutants is consistent with the *tm1036* deletion allele being a stronger loss-of-function than the *n703n1434* allele (Fig 1A), as previously reported [38]. Conversely, we found that *ces-1(n703)* gf mutants exhibit increased reversal frequencies, consistent with increased glutamatergic signaling (Fig 2A–2C). These results suggest that *ces-1* bi-directionally regulates glutamatergic signaling. We next tested if the effect of *ces-1(n703)* gf on spontaneous reversals was dependent on *glr-1* by analyzing *ces-1(n703); glr-1(n2461)* double mutants. We found that the increased reversal frequencies observed in *ces-1(n703)* gf were blocked by *glr-1* null mutants, suggesting that *ces-1* regulates spontaneous locomotion reversals in a *glr-1*-dependent manner (Fig 2B).

### *ces-1* regulates spontaneous locomotion reversals independent of *ces-2*

We next tested whether the effects of *ces-1* on spontaneous reversals were indirectly due to its known role in regulating cell death in the NSM lineage. CES-2 is a bZIP transcription factor that binds to the upstream regulatory region of *ces*-1 and negatively regulates CES-1 expression and function in the NSM lineage [35, 37, 64]. Like *ces-1* gf mutations, *ces-2* lf mutations block the cell death of NSM sister cells [35, 40]. If the increase in spontaneous locomotion reversals observed in *ces-1* gf mutants is due to additional NSM cells, then we would expect *ces-2* lf mutants to exhibit a similar phenotype. However, we found that a putative null allele which is known to block NSM sister cell deaths, *ces-2*(*bc213*) [37], and another independent *ces-2* lf allele, *gk1020*, exhibit wild-types frequencies of spontaneous reversals (Fig 2C). These results indicate that *ces-2* does not regulate glutamatergic behavior and suggests that the mechanism by which *ces-1* regulates glutamatergic behavior is independent of *ces-2* and thus distinct from its known role in regulating cell death in the NSM lineage.

### *ces-1* mutants have normal numbers of GLR-1-expressing neurons

**Fig 3:**
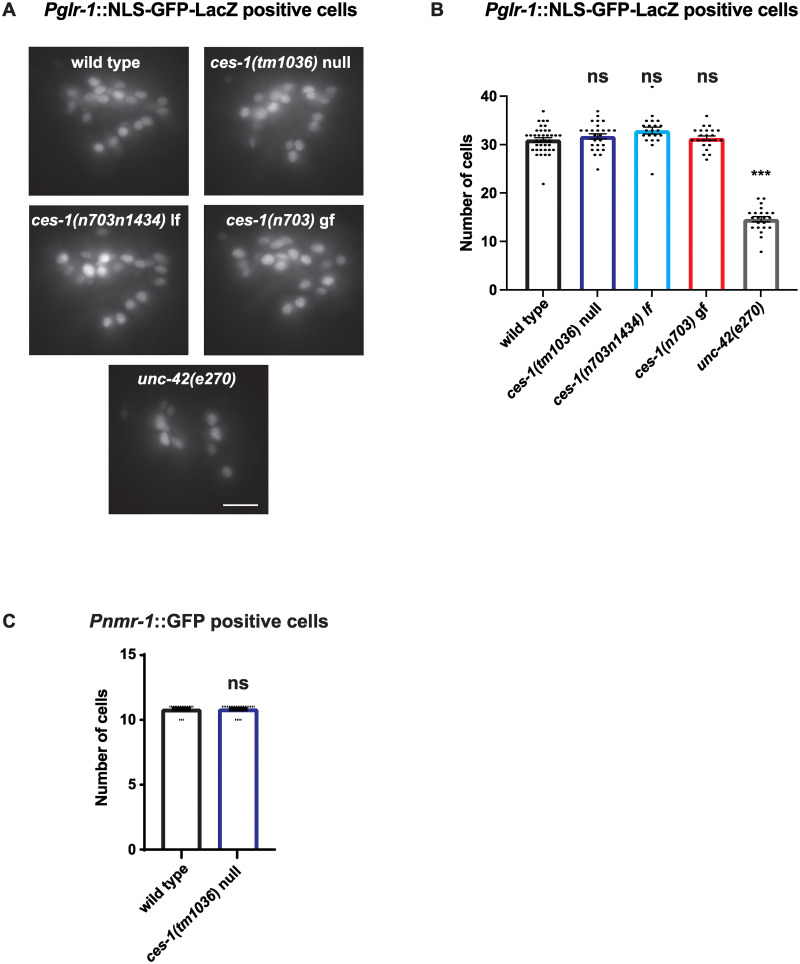
The number of GLR-1::GFP positive head neurons is unaltered in *ces-1* mutants. (A) Representative images of GLR-1 positive head neurons (*Pglr-1*::*nls*::*GFP*::*lacZ)* in wild type, *ces-1*, and *unc-42* mutant backgrounds. (B) Quantitation of GLR-1 positive head neurons in (A). N = 38 animals for wild type, N = 26 for *ces-1(tm1036)*, N = 21 for *ces-1(n703n1434)*, N = 23 for *ces-1(n703)*, and N = 22 for *unc-42*. (C) Quantitation of NMR-1 positive head neurons (*Pnmr-1*::*GFP*). N = 17 animals for wild type and N = 23 animals for *ces-1(tm1036)*. Scale bar represents 10 micrometers. Error bars represent SEM. “ns” indicates no statistically significant difference from wild type (p>0.05). One-way ANOVA with Dunnett’s multiple comparisons test was used to compare means. ***p<0.0005.

Because ablation of GLR-1-expressing command interneurons reduces the nose-touch response and spontaneous reversal frequency [21, 60], we tested whether *ces-1* mutations result in ectopic cell death of GLR-1-expressing neurons. We counted the number of GLR-1-expressing cell bodies in the head using a GFP reporter under control of the *glr-1* promoter (*Pglr-1*::NLS-GFP-LacZ (*pzIs29)*) [43]. As expected, we found fewer GLR-1-expressing cells in animals with mutations in the transcription factor *unc-42*, which is known to regulate the differentiation of GLR-1-expressing neurons [65, 66] (Fig 3A and 3B). We found no change in the number of GLR-1-expressing neurons in *ces-1(tm1036)* lf, *ces-1(n703n1434)* lf, or *ces-1(n703)* gf mutant animals (Fig 3A and 3B). We also counted neurons using another independent reporter, *Pnmr-1*::*gfp* (*akIs3*), that is expressed in a smaller subset of GLR-1-expressing neurons (11 cells) allowing us to definitively identify and count the backward command interneurons involved in locomotion, AVA, AVD, and AVE. Consistent with our previous results, we found no change in the number of this subset of neurons in *ces-1*(*tm1036*) null animals (Fig 3C). Overall, these results suggest that the effect of *ces-1* on glutamatergic behaviors is not due to ectopic cell death of GLR-1-expressing neurons.

### GLR-1 expression is not reduced in *ces-1* mutants

**Fig 4:**
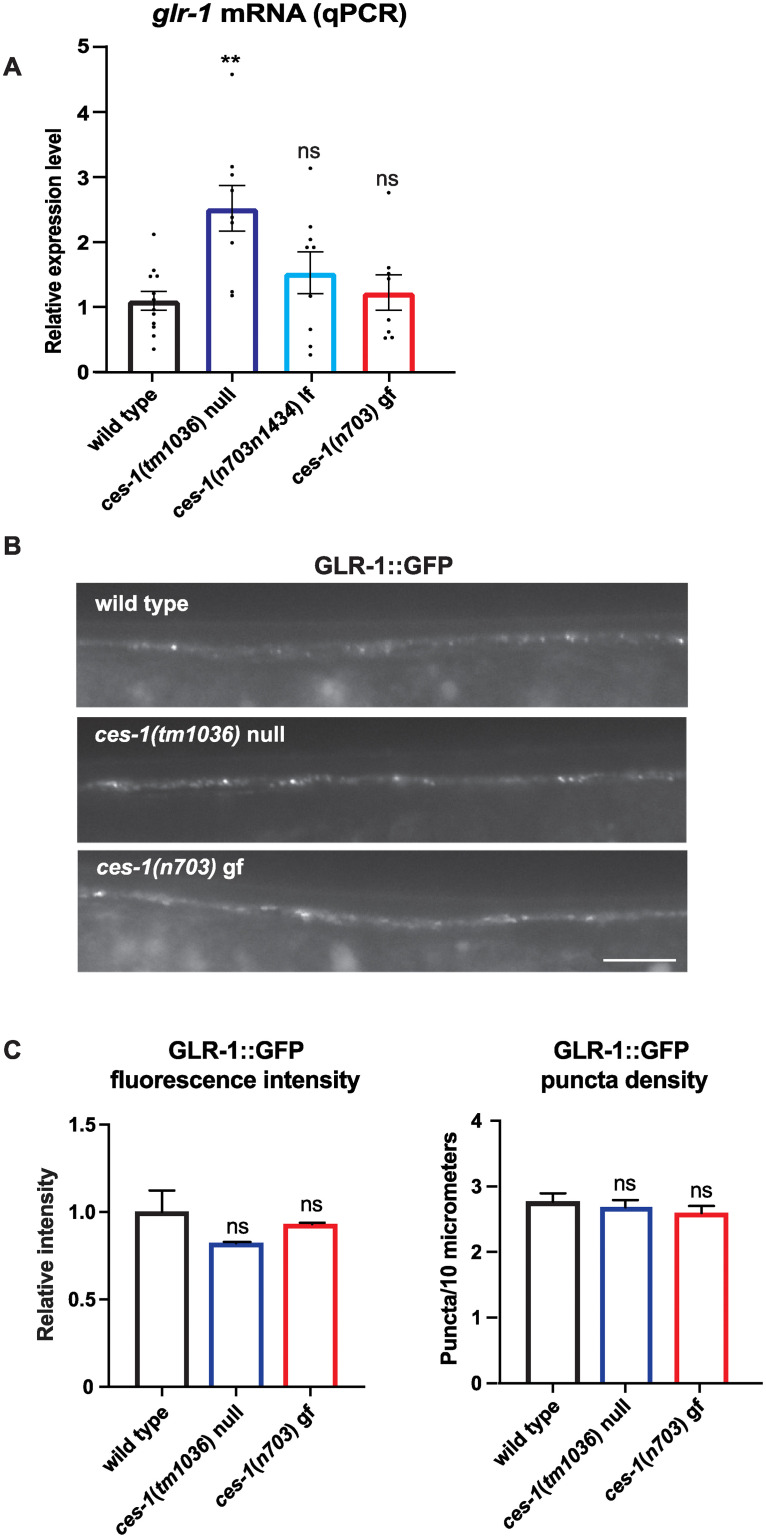
*glr-1* mRNA and GLR-1::GFP levels are not decreased in *ces-1* loss-of-function mutants. (A) Real-time qPCR for *glr-1* mRNA in wild type and *ces-1* mutants. Data represents 4 experiments and 3 biological replicates normalized to 2 reference genes (ΔΔCt versus *act-1* and *ama-1)*. (B) Representative images of GLR-1::GFP (*pzIs12)* in the ventral nerve cord of wild type and *ces-1* mutants. (C) Quantitation of peak fluorescence intensity (Normalized) and density of GLR-1::GFP puncta in the ventral nerve cord in wild type and *ces-1* mutants. N = 56 animals for wild type, N = 28 for *ces-1(tm1036)*, and N = 31 for *ces-1(n703)*. Error bars represent SEM. “ns” indicates no statistically significant difference from wild type (p>0.05). Asterisks above the bar indicate values that differ significantly from wild type (**p<0.005). One-way ANOVA with Dunnett’s multiple comparisons test was used to compare means in (A). Tukey-Kramer test was used to compare means in (C).

Since our rescue experiment suggests that *ces-1* may act in part by functioning in GLR-1-expressing neurons to regulate the nose-touch response (Fig 1B), we tested if *ces-1* regulates GLR-1 expression. We first investigated whether *glr-1* mRNA levels were decreased in *ces-1* lf mutants using RT-qPCR. We found that *glr-1* mRNA levels (normalized to *act-1* and *ama-1* reference genes) were not reduced, but were instead increased in *ces-1 (tm1036)* null mutants (Fig 4A). *glr-1* mRNA levels were unaltered in *ces-1(n703n1434)* lf and *ces-1(n703)* gf mutants. This result is not consistent with CES-1 directly promoting *glr-1* transcription to increase GLR-1-dependent behavior because *ces-1* lf mutants have decreased glutamatergic behaviors and would thus be predicted to have decreased *glr-1* expression. Instead, the increased *glr-1* mRNA levels observed in *ces-1* lf mutants is consistent with a negative feedback loop where decreased GLR-1 signaling triggers a compensatory increase in *glr-1* transcription, as we previously described [43].

We next tested whether CES-1 regulates GLR-1 protein levels and distribution by analyzing the puncta fluorescence intensity and density of GFP-tagged GLR-1 (GLR-1::GFP) expressed under the control of the *glr-1* promoter (*pzIs12*). Expression of GLR-1::GFP in the ventral cord interneurons rescues the nose-touch defect observed in *glr-1* null mutants, indicating that the tagged receptor is functional [67]. We found that GLR-1::GFP [18, 19] puncta fluorescence levels and density were unchanged in the ventral nerve cord (VNC) of both *ces-1* lf and gf mutants compared to wild-type controls (Fig 4B and 4C). Collectively, these data suggest that the defects in glutamatergic behavior observed in *ces-1* mutants is likely not due to a reduction in GLR-1 expression.

### *ces-1* mutants suppress a constitutively-active form of GLR-1

**Fig 5:**
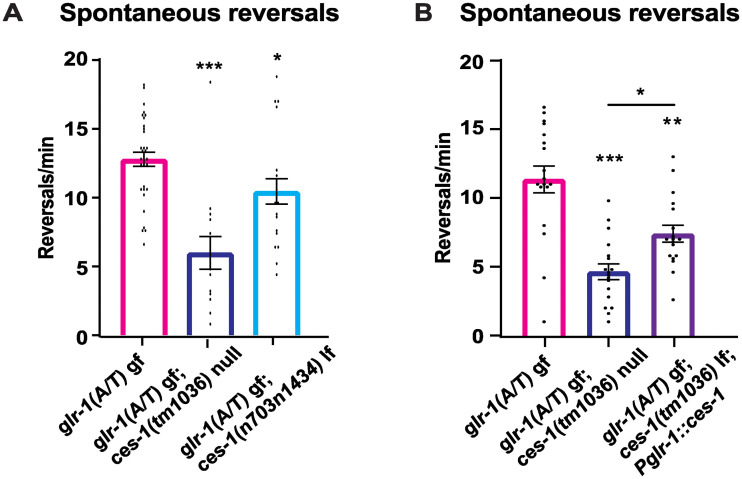
*ces-1* mutants suppress the behavioral effects of a constitutively-active GLR-1. Spontaneous reversal frequency measured for *glr-1* (A/T) gf and *glr-1* (A/T) gf; *ces-1* lf mutants. N = 34 animals for *glr-1(A/T)*, N = 14 for *glr-1(A/T); ces-1(tm1036)*, and N = 20 for *glr-1(A/T); ces-1(n703n1434)* in (A). N = 18 animals each for *glr-1 (A/T)*, *glr-1(A/T); ces-1(tm1036)*, and *glr-1(A/T); ces-1(tm1036); Pglr-1*::*ces-1* in (B). Error bars represent SEM. Asterisks above the bar indicate values that differ significantly from wild type. One-way ANOVA with Dunnett’s multiple comparisons test was used to compare means. *p<0.05, **p<0.005, ***p<0.0005.

Since *ces-1* lf mutants have a reduction in glutamatergic behaviors without a correlate decrease in GLR-1 expression, we tested whether *ces-1* alters GLR-1 signaling by analyzing the effects of *ces-1* lf mutants on a constitutively-active form of GLR-1, GLR-1(A/T)::YFP, expressed in GLR-1-expressing neurons (*nuIs80*). Mutation of a conserved alanine to a threonine in the pore domain of mouse GluRδ2(A/T) or *C*. *elegans* GLR-1(A/T) results in a gain-of-function, constitutively-active receptor [60, 68]. Expression of GLR-1(A/T) in the command interneurons results in an increased frequency of spontaneous reversals [60], consistent with increased glutamatergic signaling. We found that both *ces-1* lf alleles were able to suppress the increased reversals, again with the *tm1036* allele having a stronger effect than the *n703n1434* allele (Fig 5A), as previously described [38]. Expression of wild type *ces-1* in GLR-1-expressing neurons (using the *glr-1* promoter) partially rescued this suppression (Fig 5B). The ability of *ces-1(tm1036)* to suppress the effects of GLR-1(A/T)::YFP on reversal frequency is not due to reduced expression of the transgene because the fluorescence intensity levels of GLR-1(A/T)::YFP in the VNC of *ces-1(tm1036)* mutants are comparable to wild type (GLR-1(A/T)::YFP Fluorescence Intensity Mean ± SEM (Norm.): WT: 102.1 ± 6.5; *ces-1* (*tm1036*): 115.8 ± 8.8; n = 23–24 animals per genotype. n.s. p>0.05, Student’s *t* test). Overall, these data suggest that CES-1 functions, in part, by acting in GLR-1-expressing neurons, and are consistent with the idea that CES-1 regulates glutamatergic behavior by promoting some aspect of GLR-1 function or signaling.

## Discussion

We identified *ces-1* Snail in an RNAi screen for conserved transcription factors that regulate glutamatergic behavior. The Snail family of zinc finger transcription factors are conserved from *C*. *elegans* to mammals and regulate a variety of processes, including cell polarity, cell proliferation, lineage commitment, and cell death during development [22, 23, 30]. *ces-1* was originally discovered as a cell death regulator in *C*. *elegans*, where it is known to selectively inhibit the death of cells in just two lineages [35, 40]. Specifically, *ces-1* gf mutants block the programmed cell death of NSM and I2 sister cells. *ces-1* lf mutants do not have any obvious cell death phenotypes in these cells [40]. However, *ces-1(tm1036)* lf mutants affect asymmetric cell division and proliferation of the cells that give rise to the NSM and the NSM sister cells, the NSM neuroblasts [36, 38]. In this study, we identify a novel role for *ces-1* in regulating glutamatergic behavior that appears to be independent of its canonical role in regulating the cell death of NSM sister cells.

Although *ces-1* has almost exclusively been studied in cells of the NSM lineage, several studies hint at functions for *ces-1* in other lineages. For example, *ces-1* has been reported to be expressed in other unidentified lineages beyond the NSM lineage [37]. *ces-1* can act together with the cell cycle regulatory genes, *cdc-25*.*2* phosphatase and *cya-1* cyclin, to regulate cell proliferation in several other cell lineages [36]. Lastly, analysis of transcriptional regulatory networks in diverse tissues using large-scale yeast one-hybrid assays identified several potential *ces-1*-regulated genes containing predicted CES-1 Snail binding E-box motifs [69]. Analysis of *ces-1* lf mutants revealed that the expression of one of these genes, PB0507.1, was eliminated in the pharyngeal intestinal valve, spermathecal, distal tip cell, and rectal gland, providing further evidence that CES-1 can regulate genes outside the NSM lineage [69]. Our rescue data suggest that *ces-1* may function in part by acting in GLR-1-expressing neurons to regulate behavior. Although we were not able to detect *ces-1* expression in GLR-1-expressing neurons between the L1 larval stage and adults using a single-copy *ces-1* reporter transgene (*bcSi66*, *Pces-1*::*ces-1*::*mNeonGreen*) [39], *ces-1* is expressed in many unidentified cells in the embryo. Interestingly, a recent large-scale transcriptome study using *C*. *elegans* embryos was able to detect *ces-1* transcripts in *glr-1*-expressing neurons including the backward command interneurons AVA and AVE [70]. However, since we did not detect expression of a *ces-1* translational reporter (*bcSi66*) in larva or adults, it is possible that *ces-1* expression may be low in *glr-1*-expressing neurons, controlled by distal regulatory elements not included in the transgene or upregulated in response to certain stimuli. Consistent with this notion, while *ces-1* is known to act in NSM cells to repress death, expression of another *ces-1* reporter construct (*bcIs58*, *Pces-1*::*ces-1*::*yfp*) was only detected in 2 out of 17 NSM cells examined [37]. However, in *ces-2* or *dnj-11* mutants, CES-1::YFP expression was detected in NSMs, NSM sister cells and NSM neuroblasts, which suggests that CES-2 and DNJ-11 repress *ces-1* expression in these cells but also suggests that *ces-1* expression may be normally kept at low levels and could potentially be expressed under certain conditions or developmental stages [37].

We found that *ces-1* lf mutants have defects in two glutamatergic behaviors: the mechanosensory nose-touch response (Fig 1) and spontaneous locomotion reversals (Fig 2). The finding that *ces-1* mutants have defects in spontaneous reversals in addition to the nose-touch response suggests that *ces-1* mutants affect the signaling involved in at least two different glutamatergic behaviors. ASH sensory neurons are involved in the nose-touch response, and cell ablation of ASH results in a defect in the nose-touch response [16] with no effect on the frequency of spontaneous locomotion reversals [71]. Thus, the spontaneous reversal defect observed in *ces-1* mutants cannot be attributed to a selective impairment in ASH-dependent glutamate signaling.

The defects in the nose-touch and spontaneous reversal behaviors are not due to alterations in NMJ or muscle function because *ces-1* mutants have wild type NMJ function based on the aldicarb-paralysis assay (Fig 1C). Additionally, because the *C*. *elegans* NMJ is regulated by both cholinergic and GABAergic signaling and defects in either alter the rate of aldicarb-induced paralysis [59, 72], these data suggest that *ces-1* mutants do not have a general defect in synaptic transmission, but rather a relatively specific defect in glutamatergic signaling.

*ces-1* gf mutants are known to block the cell death of NSM and I2 interneuron sister cells during development. Because cell ablation of ASH results in nose touch defects [16], we tested whether *ces-1* lf mutants have defects in the nose-touch response due to ectopic cell death of ASH sensory neurons. We found that *ces-1* lf mutants have normal numbers of ASH sensory neurons based on a YFP reporter expressed in ASH. Backfilling the ASH sensory neuron dendrite and cell body from the tip of the nose with the fluorescent lipophilic DiI dye confirmed these results and additionally suggested there were no gross ASH dendrite development defects. Furthermore, it is unlikely that the ASH dendrite or sensory transduction are defective in the absence of *ces-1* because RNAi knockdown of *ces-1* results in defects in the optoASH assay, which suggests that the defect is located somewhere downstream of ASH activation and sensory transduction. Because cell ablation of GLR-1-expressing command interneurons results in defects in the nose-touch response and in spontaneous reversal behavior [21, 60], we also tested if *ces-1* lf mutants result in the ectopic cell death of GLR-expressing neurons. Our data show that the number of GLR-1-expressing neurons, including the number of command interneurons AVA, AVD and AVE involved in backward locomotion in response to nose-touch stimuli and during spontaneous locomotion reversals, is unchanged in *ces-1* mutants (Fig 3). Collectively, these data suggest that the glutamatergic behavior defects observed in *ces-1* mutants is not due to alterations in the number of ASH sensory neurons or GLR-1 expressing interneurons, suggesting that *ces-1* does not regulate cell proliferation or cell death of these cells.

Our data also suggest that the effect of *ces-1* on glutamatergic behavior is independent of its canonical role in regulating cell death in the NSM lineage. In NSM sister cells, the transcription factor CES-2 negatively regulates expression of CES-1 to control cell death [35, 40]. *ces-2* lf mutants phenocopy *ces-1* gf mutants, resulting in survival of the NSM sister cells. While *ces-1* gf mutants have increased spontaneous reversals, two independent *ces-2* lf alleles, including the *bc213* allele previously shown to block NSM sister cell death [38], have no effect on spontaneous reversal frequency (Fig 2). These data show that the role of *ces-1* on glutamatergic behavior is independent of *ces-2* and thus likely independent of its role in regulating NSM sister cell death.

The defects in glutamatergic behavior observed in *ces-1* mutants were partially rescued by expressing wild type *ces-1* cDNA in *glr-1*-expressing neurons (Figs 1B and 5B), suggesting that *ces-1* functions, at least in part, by acting in *glr-1*-expressing neurons to regulate behavior. This partial rescue implies that *ces-1* may also act in other cell types to indirectly regulate glutamatergic behavior. One possibility is that *ces-1* regulates spontaneous reversal behavior by altering serotonin signaling. Increased serotonin signaling promotes a feeding behavior known as dwelling where the worm changes direction frequently to remain on a patch of food [73]. Since NSM is a serotonergic neuron, *ces-1* is known to function in the NSM lineage, and *ces-1* gf mutants promote survival of NSM sister cells, it is possible that the increased spontaneous reversals that we observe in *ces-1* gf mutants may be attributed in part to alterations in serotonin signaling. While this model is possible, it is unlikely that the increased spontaneous locomotion reversals observed in *ces-1* gf mutants is due to increased NSM sister cell survival because we found that *ces-2* lf mutants, which like *ces-1* gf mutants promote increased NSM sister cell survival [35, 40], do not exhibit increased spontaneous reversals (Fig 2C). Future experiments will be required to investigate the relationship between spontaneous locomotion reversal frequency, which is assayed in the absence of food, and the roaming and dwelling locomotion feeding behavior, which is assayed in the presence of food, and the potential interplay between *ces-1*, glutamate and serotonin signaling in these behaviors.

Since loss of *glr-1* can also result in defects in nose touch and spontaneous reversals [18, 19, 44, 62], we analyzed whether *glr-1* was a transcriptional target of CES-1 by testing whether *ces-1* lf mutants had decreased *glr-1* expression. We found no reduction in *glr-1* mRNA, but instead observed an increase in *glr-1* mRNA in *ces-1(tm1036)* mutants (Fig 4A). This increase is consistent with a negative feedback pathway we previously characterized, where decreases in GLR-1 function result in a compensatory feedback signal via CMK-1/CaM Kinase to increase *glr-1* transcription [43]. This data suggests that *glr-1* may not be a direct transcriptional target of CES-1 in these neurons since the behavioral defects are not consistent with increased *glr-1* expression.

The direct transcriptional target of CES-1 in *glr-1* neurons that promotes glutamatergic behaviors remains to be determined. Wei et al. (2017) identified 3,199 genes as potential transcriptional targets of CES-1 based on chromatin immunoprecipitation-sequencing data from the modENCODE project [38, 74] Interestingly, our analysis of this data set revealed 32 potential CES-1 target genes that have been shown to regulate GLR-1. Several of these genes are known to either positively or negatively regulate GLR-1 trafficking, endocytosis or recycling, including the clathrin adaptors *unc-11*/AP180 [52] and the AP2 complex genes [75], the endocytic adaptor *ehbp-1* [76], the recycling GTPase *rab-10* [77] and several ubiquitin system genes including the ubiquitin ligase subunits *rpm-1*, *dlk-1* [78], *emb-27* [62], *kel-8* and *cul-3* [63], and *wdr-20*, an activator of the deubiquitinating enzyme USP-46 [61]. CES-1 has traditionally been shown to act as a transcriptional repressor, however one report suggests that CES-1 may possibly act as a transcriptional activator [69]. Thus, depending on whether CES-1 acts an activator or repressor, one could propose models for how CES-1 might control genes known to regulate GLR-1 trafficking. For example, since loss of the clathrin adpatin *unc-11*/AP180 leads to accumulation of GLR-1 at the neuronal surface [52], we speculate that CES-1 may repress expression of *unc-11* and inhibit GLR-1 endocytosis. Interestingly, two genes required for GLR-1 function, the glutamate receptor auxiliary subunits *sol-1* and *stg-2* [79, 80], were also identified in the data set of potential CES-1 targets [38]. In this case, we speculate that CES-1 could promote GLR-1 function by activating expression of *sol-1* and *stg-2*. Although we cannot distinguish between surface and internal pools of receptor, our analysis of GLR-1::GFP levels in the VNC suggest that *ces-1* mutants do not alter total GLR-1 expression or distribution in the VNC. However, it will be interesting in the future to test if the expression of these genes known to regulate GLR-1 are directly controlled by CES-1 and whether the cell surface levels or function of GLR-1 are altered in *ces-1* mutants.

Our data show that *ces-1* lf mutants suppress the effects of constitutively-active GLR-1(A/T) on behavior, suggesting that *ces-1* may regulate some aspect of GLR-1 function. Because the effects of GLR-1(A/T) are independent of presynaptic glutamate release [60], the ability of *ces-1* to suppress the effects of GLR-1(A/T) on reversal behavior suggests that the *ces-1* mutant defects are not likely due to an alteration in presynaptic glutamate release. Although our rescue data suggest that CES-1 may act in part by functioning in GLR-1 expressing neurons, a lack of direct evidence showing that *ces-1* is expressed in these neurons in larva or adults leaves open the possibility that CES-1 regulates cell death or development of these or other cells that affect the development of *glr-1*-expressing neurons and glutamatergic behavior. Because *ces-1* is involved in cell fate and cell polarity and also appears to be expressed outside the NSM cell lineage, it remains formally possible that *ces-1* mutants have an effect on cell death or development in other cells that we did not examine, which may indirectly affect glutamatergic behavior. Our analysis of GLR-1::GFP distribution in the VNC suggests that there are no obvious abnormalities in the number of GLR-1-containing puncta, suggesting that development of GLR-1-expressing neurons in the VNC appears grossly normal. Nevertheless, our data are consistent with the idea that *ces-1* directly or indirectly affects some aspect of GLR-1 function. Given the list of potential CES-1 target genes that are known to regulate GLR-1 signaling or trafficking (i.e., genes that affect cell surface levels of GLR-1), we speculate that *ces-1* regulates glutamatergic behavior, in part, by altering GLR-1 signaling or controlling levels of GLR-1 at the synaptic surface. Future studies will be required to determine the precise mechanism by which *ces-1* regulates glutamatergic behavior. In conclusion, this study identifies a novel role for *ces-1* in regulating glutamatergic behavior independent of its canonical role in regulating cell death in the NSM lineage.

## Supporting information

S1 Fig Cumulative Probability Histograms of GLR-1::GFP puncta (A) fluorescence intensities and (B) densities. Related to Fig 2C.
(PDF)

S1 Table Aldicarb-paralysis data. Related to Fig 1C.
(XLSX)
