# Supplementary figures and images for: The Snail transcription factor CES-1 regulates glutamatergic behavior in C. elegans
Source: PLoS One. 2021 Feb 2;16(2):e0245587. doi: 10.1371/journal.pone.0245587 (PMC7853468; doi:10.1371/journal.pone.0245587)

S1 Fig

A

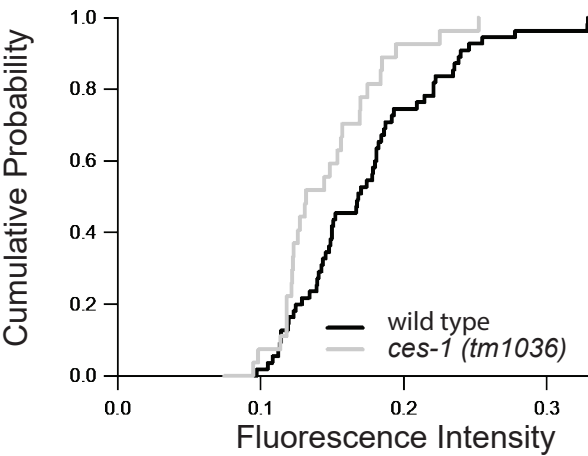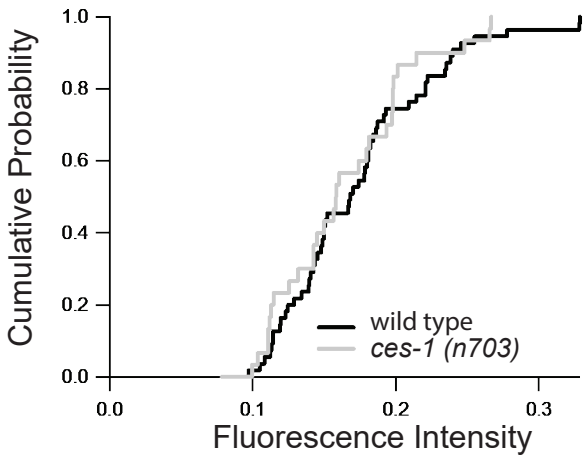

B

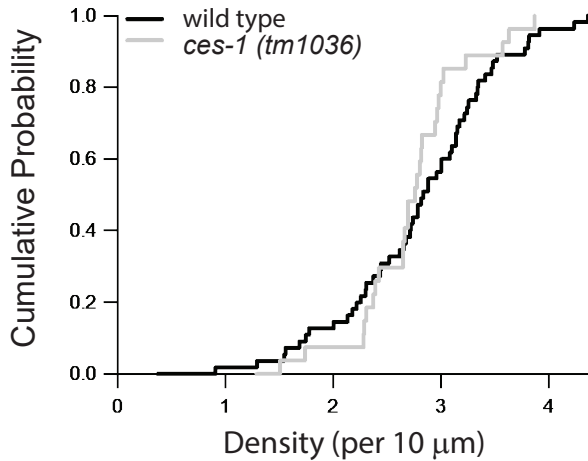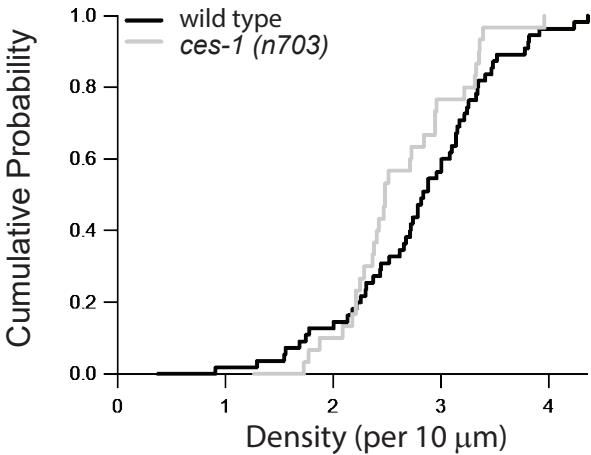

Supplement: S1 Fig — Related to Fig 2C. (PDF) [file pone.0245587.s001.pdf]
